# Factors Related To Retention Of Foreign “Specified Skilled Nursing Care Workers” In Japan

**DOI:** 10.1093/geroni/igaf122.3026

**Published:** 2025-12-31

**Authors:** Noriko Tsukada, Jun Nishimura

**Affiliations:** Nihon University, Tokyo, Tokyo, Japan; Rissho University, Tokyo, Tokyo, Japan

## Abstract

The Japanese government created a new residence status, “Specified Skilled Workers,” to recruit foreign workers in 2019 to cope with serious labor shortages in the 14 industries, including nursing care. There is no restriction on the countries eligible to enter Japan. This study examined factors related to the retention of Specified Skilled nursing care (SSNC) workers. Structured questionnaires were sent to randomly selected 1000 corporations/hospitals nationwide that hired SSNC workers. 9 questionnaires were sent to each address, and SSNC workers were asked to respond to the survey electronically from October 31 to November 30, 2024. Despite the low effective response rate(4%), a total of 340 responses were analyzed in this study. About 93% of the respondents came from 5 countries, including Vietnam(34.1%), Philippines(25.3%), Indonesia(14.1%), Myanmar(10%) and Nepal(9.4%). Preliminary bivariate analyses revealed that “levels of desire to obtain national nursing care certificate” and “job satisfaction” were found to be statistically significantly related to retention of SSNC workers; however, its strength of the relationships varied among countries. For example, as for the levels of desire to obtain a national certificate, while it was the highest(r=.807**) for Philippine SSNC workers, it was the lowest(r=.361*) for Nepal SSNC workers. Moreover, among 37 factors asked, 11 factors were statistically significantly related to retention for Myanmar SSNC workers, while for Indonesians one factor, “increase the number of retakes of national exams,” was statistically significant. It is suggested that the government be aware of these differences among countries and develop country-specific-policies to promote retention of SSNC workers.

